# Stem cell therapy for COVID‐19, ARDS and pulmonary fibrosis

**DOI:** 10.1111/cpr.12939

**Published:** 2020-10-24

**Authors:** Zhongwen Li, Shuaishuai Niu, Baojie Guo, Tingting Gao, Lei Wang, Yukai Wang, Liu Wang, Yuanqing Tan, Jun Wu, Jie Hao

**Affiliations:** ^1^ Institute of Zoology State Key Laboratory of Stem Cell and Reproductive Biology Chinese Academy of Sciences Beijing China; ^2^ Institute for Stem Cell and Regeneration Chinese Academy of Sciences Beijing China; ^3^ National Stem Cell Resource Center Chinese Academy of Sciences Beijing China; ^4^ University of Chinese Academy of Sciences Beijing China

**Keywords:** clinical trials, COVID‐19, mesenchymal stem cells, stem cell therapy

## Abstract

Coronavirus disease 2019 (COVID‐19) is an acute respiratory infectious disease caused by severe acute respiratory syndrome coronavirus 2 (SARS‐CoV‐2). COVID‐19 mainly causes damage to the lung, as well as other organs and systems such as the hearts, the immune system and so on. Although the pathogenesis of COVID‐19 has been fully elucidated, there is no specific therapy for the disease at present, and most treatments are limited to supportive care. Stem cell therapy may be a potential treatment for refractory and unmanageable pulmonary illnesses, which has shown some promising results in preclinical studies. In this review, we systematically summarize the pathogenic progression and potential mechanisms underlying stem cell therapy in COVID‐19, and registered COVID‐19 clinical trials. Of all the stem cell therapies touted for COVID‐19 treatment, mesenchymal stem cells (MSCs) or MSC‐like derivatives have been the most promising in preclinical studies and clinical trials so far. MSCs have been suggested to ameliorate the cytokine release syndrome (CRS) and protect alveolar epithelial cells by secreting many kinds of factors, demonstrating safety and possible efficacy in COVID‐19 patients with acute respiratory distress syndrome (ARDS). However, considering the consistency and uniformity of stem cell quality cannot be quantified nor guaranteed at this point, more work remains to be done in the future.

## Graphical abstract

The potential mechanisms of MSCs therapy for COVID‐19. MSCs have great therapeutic potential in immunomodulation and tissue repair through secretion of soluble paracrine protein factors and exosomes. MSCs can regulate the functions of a variety of immune cells, secrete several cytokines, promote tissue repair and regeneration, and may play important therapeutic roles in patients with COVID‐19. MSCs: mesenchymal stem cells; HGF, hepatocyte growth factor; VEGF, vascular endothelial growth factor; KGF, keratinocyte growth factor; FGF, fibroblast growth factor; TGF‐β, transforming growth factor‐β; TNF‐α, tumor necrosis factor‐α; MSC‐exo, exosomes.

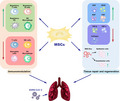

## 1 INTRODUCTION

In December 2019, an outbreak of unidentifiable pneumonia cases was first officially reported in Wuhan, China. It was subsequently confirmed that the pneumonia is an acute respiratory infectious disease caused by infection of severe acute respiratory syndrome coronavirus 2 (SARS‐CoV‐2), a novel β‐coronavirus which had never been reported before.^1^, ^2^ As the global epidemic grew and spread rapidly, the World Health Organization (WHO) officially named the new type of disease as coronavirus disease 2019 (COVID‐19). COVID‐19 was confirmed to be more contagious than either the severe acute respiratory syndrome (SARS) or Middle East respiratory syndrome (MERS), with a confusing manifestation ranging from asymptomatic patients to severely ill patients with acute respiratory distress syndrome (ARDS) and pulmonary fibrosis, amongst several potential health problems, and has had disastrous consequences for public health management.

## 2 PATHOGENESIS OF COVID‐19

### 2.1 The novel coronavirus and its infection pathways

**FIGURE 1:**
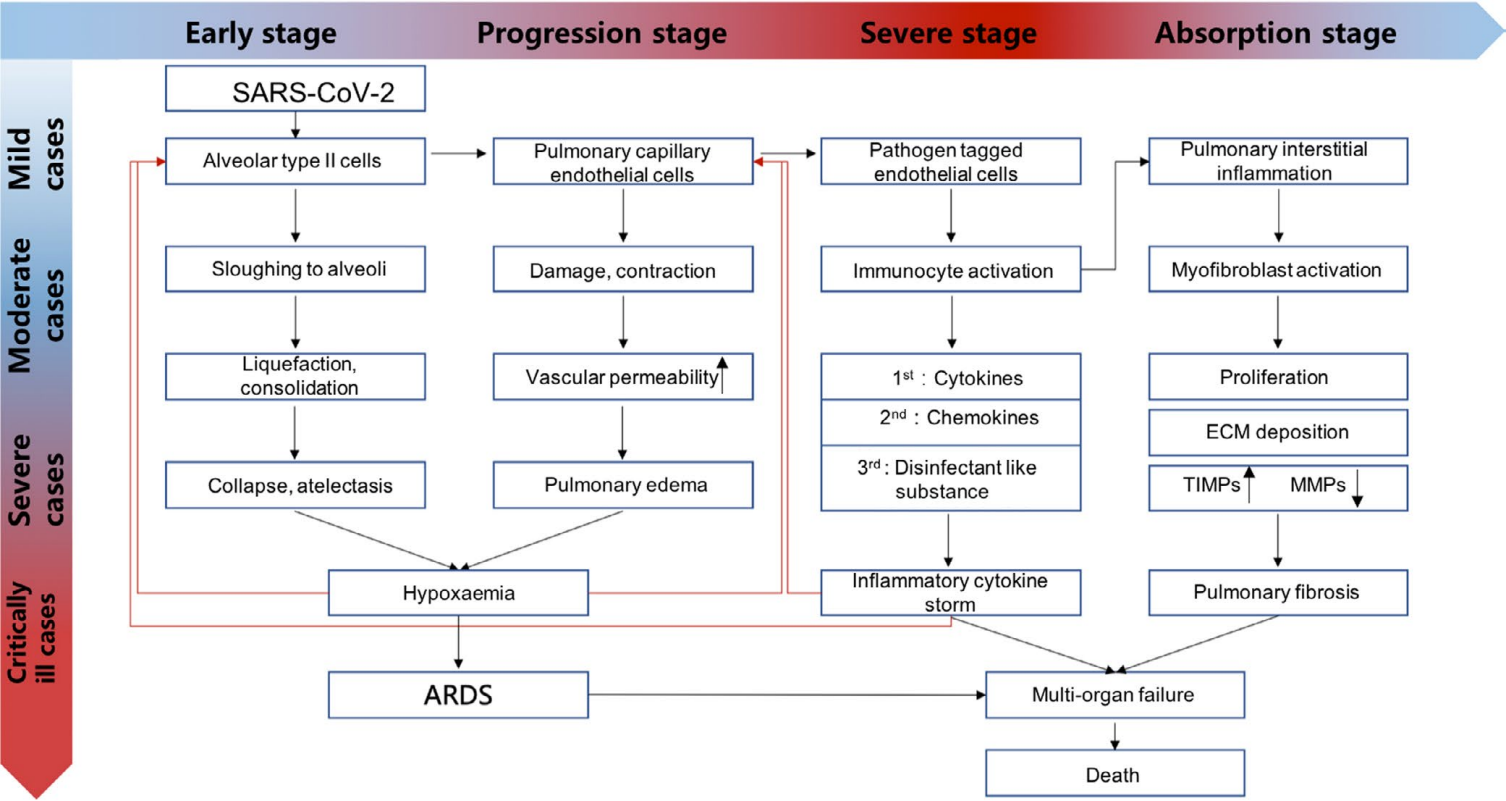
COVID‐19 pathogenic progression

Initially named 2019‐nCoV, SARS‐CoV‐2 belongs to the subfamily Coronavirinae in the family Coronaviridae of the order Nidovirales. As a single‐stranded positive‐stranded RNA virus of the β subclass of the coronavirus genus, it is only the 7th human coronavirus to be discovered. The virus has a diameter of about 80‐120 nm and a full‐length genome of approximately 29.9 kb. It can encode 29 proteins and has 79% homology to the SARS virus sequence. The spike glycoprotein (S protein) on its surface is an essential structural protein that mediates its invasion into human cells. Through the host cell receptor‐angiotensin converting enzyme 2 (ACE2), SARS‐CoV‐2 adsorbs onto and enters the host, then replicates, assembles, and releases a large number of viral particles.^3^ ACE2 is expressed on the surface membrane of alveolar, tracheal and bronchial epithelial cells in the lung, and monocytes and macrophages in the immune system. It can also be expressed in the heart, kidney and intestines. ACE2 lowers blood pressure and regulates the renin‐angiotensin system by inactivating angiotensin II (Ang II) produced by ACE, and serves as a crucial regulator of pulmonary oedema. SARS‐CoV‐2 utilizes a highly glycosylated homotrimeric S protein to enter the host cell, and its affinity for ACE2 is 10‐20 times that of SARS virus, thus enhancing its transmissibility.^4^ In fact, although the fatality rate of COVID‐19 is lower than SARS (9.6%) and MERS (34.4%), its higher infectious rate has led to a much wider outbreak with significantly more complications in epidemic prevention and control, due to a large number of asymptomatic and mild patients (Figure 1).

For patients with symptoms, the incubation period (time from exposure to onset of symptoms) has a wide range but averages to ~4‐5 days.^5^ The most common symptoms include dry cough, fever and shortness of breath. Other common symptoms are myalgia, fatigue, sore throat, nausea, vomiting, diarrhoea, conjunctivitis, anorexia and headache (cdc.gov/coronavirus/2019‐ncov/hcp/clinical‐guidance‐management‐patients.html). For a small number of severely ill patients, the disease begins to worsen about 5‐10 days after the onset of symptoms, and complications such as acute respiratory distress syndrome (ARDS) and other end‐organ failures could occur.^6^ The mortality rate is significantly higher amongst elderly adults over 65. Adults with underlying cardiovascular disease, respiratory disease, endocrine metabolic disease, diabetes or a weakened immune system are the most vulnerable to serious complications of COVID‐19.^7^

### 2.2 Cytokine release syndrome

One of the reasons for aggravated severe illness in COVID‐19 patients aggravation is the excessive immune response associated with the cytokine release syndrome (CRS), which in turn leads to lung tissue damage, repair imbalance and respiratory failure. The patient could also eventually die from multiple organ failure.^8^ CRS, also known as a 'cytokine storm', is an aberrant systemic inflammatory response that can be triggered by multiple factors such as severe infections and certain drugs. The clinical manifestation is the sharp rise of a large number of cytokines within a short time frame. Analysis of plasma cytokine levels in 41 confirmed cases of COVID‐19 in China revealed that, compared with healthy adults, ICU and non‐ICU hospitalized patients’ levels of IP‐10, MCP‐1, MIP‐1A, MIP1‐B, PDGF, TNF‐α and VEGF were significantly increased.^9^ Extremely high concentrations of IL‐6, GCSF, CRP and TNF‐α have also been recorded in COVID‐19 patients.^10^ The excessive inflammatory CRS could also promote thrombosis and deaths by thromboembolism in critically ill patients.^11^, ^12^, ^13^ At present, there is no specific treatment for CRS. In clinical practice, glucocorticoid injections for systemic immune suppression and cytokine inhibitors have been the primary methods. However, the use of glucocorticoids in viral pneumonia carries additional risks of steroid treatment sequelae such as diabetes and osteonecrosis, so there has been controversy in the academic community.

### 2.3 Acute respiratory distress syndrome

ARDS refers to acute progressive hypoxic respiratory failure caused by various pulmonary and extrapulmonary pathogenic factors other than cardiogenic.^14^ According to the Berlin definition, patients with less severe hypoxaemia (as defined by a ratio of the partial pressure of arterial oxygen to the fraction of inspired oxygen of 300 or less) are considered to have acute lung injury (ALI), and those with more severe hypoxaemia (as defined by a ratio of 200 or less) are considered to have the ARDS.^15^, ^16^ ARDS is a continuous pathological process, and its early stage is ALI. The main manifestations are sudden progressive dyspnoea, varying degrees of cough, less sputum, late cough and bloody sputum. Arterial hypoxaemia is a characteristic feature, which is irresistible to oxygen therapy. If PaCO2 increased, it indicated that the patient is in critical condition. Early chest X‐ray is often negative, and then, interstitial pulmonary oedema occurs, manifested as two lungs scattered in different sizes and fuzzy edge of the patchy increased density shadow. Pulmonary interstitial fibrosis may occur in the late stage. Multiple organ failure may occur after the disease develops.^15^

### 2.4 Pulmonary fibrosis

Although many patients who develop ARDS survive the acute phase of the disease, and might even be discharged, a large proportion of them die subsequently from progressive pulmonary fibrosis.^17^ Dysregulation of matrix metalloproteinases in the inflammatory phase of ARDS could lead to a complex combination of epithelial and endothelial damage, and thus uncontrolled fibrosis.^18^ Continuous and aberrant activation of epithelial cells could lead to cellular senescence and overactive secretion of pro‐fibrotic growth factors, chemokines, vascular inhibitors and procoagulant mediators. These factors are collectively referred to as senescence‐associated secretory phenotype (SASP) factors.^19^ SASP factors can lead to abnormal wound healing, which is characterized by a dysregulated crosstalk between epithelial cells and mesenchymal cells, and the consequent accumulation of myofibroblasts. Fibroblasts and myofibroblasts in fibrotic lungs exhibit markers of stress and senescence, including resistance to apoptosis and excessive production of extracellular matrix components.^20^ The resultant increase in matrix stiffness could affect the microenvironment and thus the crosstalk between fibroblasts and epithelial cells, resulting in irreversible damage and fibrosis.^21^

### 2.5 Pathological anatomy

Studies have found that COVID‐19 can cause multiple organ and tissue damage, especially in the respiratory system.^22^, ^23^ The tracheal and bronchial mucosa exhibited hyperaemia and increased secretions.^13^ Although the pathological characteristics of lung lesions caused by SARS‐CoV‐2 are similar to SARS, there were also significant differences. Parenchymal areas contain diffuse alveolar injury and exudative inflammation.^24^ The alveolar cavity is often filled with serum, fibrin exudate and extensive transparent hyaline membrane formations, as observed in autopsies.^22^, ^25^, ^26^, ^27^ White blood cells that infiltrate the alveoli are mainly monocytes and macrophages. Type II lung alveolar cell proliferation and focal lung cell shedding can be observed. Pulmonary interstitial fibrosis is frequently observed in cases with a long duration of the disease.

COVID‐19 can also affect multiple organs with varying degrees of acute damage. SARS‐CoV‐2 was also detected in the lymph nodes, spleen, heart, liver, gallbladder, kidney, stomach, breast, skin and testis, through qRT‐PCR‐based viral nucleic acid detection, electron microscopy and immunohistochemical staining.^23^ A study noted lesions in the lymphoid hematopoietic organs.^28^ Lymphocytes in the spleen and lymph nodes, especially CD4^+^ and CD8^+^ T cells, were significantly reduced. Lymphocyte degeneration, necrosis and macrophage proliferation were frequently observed. The myocardium also exhibits cellular degeneration, occasional necrosis, interstitial oedema, and mild infiltration of monocytes, lymphocytes and/or neutrophils. Hepatocyte degeneration, spot necrosis, and small, bridging or large necrosis of neutrophil infiltration are found in the liver. In the kidney, hyperaemia, segmental hyperplasia or necrosis, and protein exudation in the glomerulus were observed. Sometimes pancreatic islet cell degeneration and lysis are detected. The oesophagus, stomach and intestinal mucosal epithelium showed varying degrees of degeneration, necrosis and exfoliation. The testes also showed different degrees of reduction and damage of spermatogenic cells. Brain congestion and oedema, some neuronal degeneration, and ischaemic changes were also detected.

## 3 POTENTIAL MECHANISMS OF STEM CELL THERAPY IN COVID‐19

**FIGURE 2:**
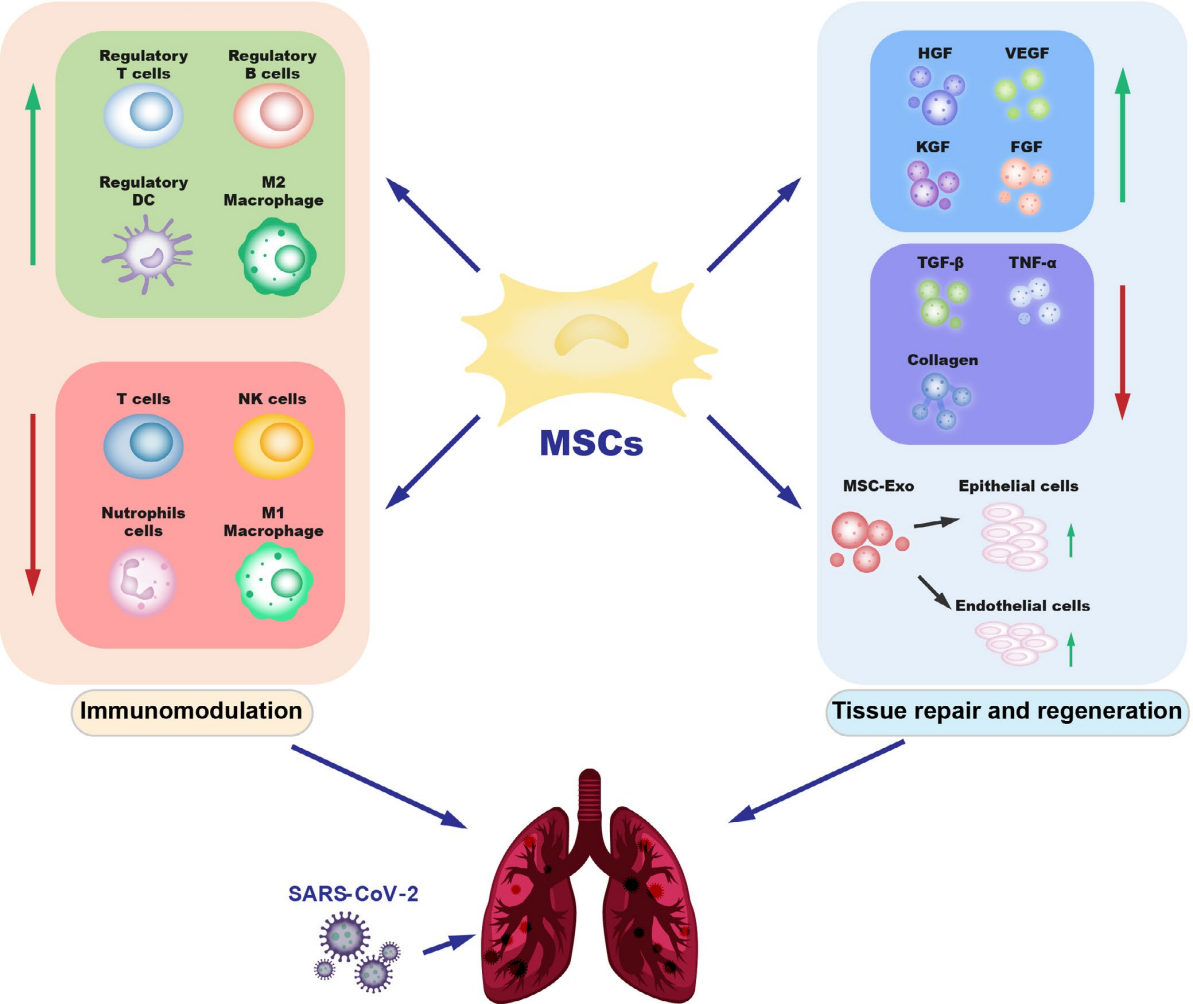
The potential mechanisms of MSCs therapy for COVID‐19. MSCs have great therapeutic potential in immunomodulation and tissue repair through secretion of soluble paracrine protein factors and exosomes. MSCs can regulate the functions of a variety of immune cells, secrete several cytokines, promote tissue repair and regeneration, and may play important therapeutic roles in patients with COVID‐19. MSCs: mesenchymal stem cells; HGF, hepatocyte growth factor; VEGF, vascular endothelial growth factor; KGF, keratinocyte growth factor; FGF, fibroblast growth factor; TGF‐β, transforming growth factor‐β; TNF‐α, tumour necrosis factor‐α; MSC‐exo, exosomes

Stem cells are endowed with the properties of self‐renewal and multi‐lineage differentiation potential, thus making them an attractive modality for cell therapy in the clinic. However, due to many ethical and legal restrictions, clinical development and progression of stem cell therapies have been relatively slow.^29^ Because adult stem cells are exempt from the aforementioned ethical and legal restrictions, while possessing excellent tissue repair capabilities, usage of adult stem cells has been more popular than embryonic or pluripotent stem cells in the clinic.^30^ Accumulating studies have shown that stem cell therapy is becoming one of the emerging treatment strategies for several refractory diseases with no known treatments, including viral infections.^31^ Newly emerging viral pandemic, which could cause multi‐organ damage and for which there are no particular therapies, drugs or vaccines available, is especially amenable to stem cell therapy. With the COVID‐19 pandemic, stem cell therapies and especially mesenchymal stem cell (MSC)‐related therapies have demonstrated their therapeutic potential for newly emerging diseases with no available treatments (Figure 2).

### 3.1 MSC‐related cells

MSCs are derived from the mesoderm and ectoderm of early embryonic development. They express specific cell surface markers such as CD73, CD90, CD105, CD29, CD44, CD146 and CD166, while being negative for CD45, CD31 and CD34.^32^, ^33^, ^34^ MSCs are also known as mesenchymal stromal cells, and these matrix‐derived cells are capable of self‐renewal and differentiation into chondrocytes, osteoblasts and adipocytes.^35^ MSCs were initially found and isolated from the bone marrow (BM), but were subsequently also discovered in various tissues such as the adipose fat pads, dental pulp, umbilical cord and placenta.^36^ Currently, MSCs from different tissues are being tested for their therapeutic effects in COVID‐19.^37^

MSCs express low levels of human leucocyte antigen (HLA) class I molecules, and do not express HLA class II molecules or costimulatory molecules such as CD40, CD40L, CD80 and CD86. This expression profile allows MSCs to escape the cytotoxic effects of lymphocytic T cells, B cells and NK cells, were thus termed 'immune‐privileged' cells.^38^, ^39^, ^40^, ^41^ In addition, MSCs possess immunomodulatory and anti‐inflammatory effects, and can detect microenvironmental injury signals to direct pro‐regenerative signalling processes,^42^, ^43^, ^44^ thus making them attractive candidates for therapeutic use in various diseases. For clinical allotransplantation, their hypo‐immunogenicity and short lifespan in vivo make them especially suitable for clinical research. Therefore, MSCs are a promising tool for the treatment of disorders involving immune dysregulation and extensive tissue damage, as is the case with COVID‐19, and multiple clinical trials have been launched.^45^

Immunity‐ and matrix‐regulatory cells (IMRCs) are a new type of hESC‐derived MSC‐like cells that resemble MSCs in their capacity for self‐renewal and tri‐lineage mesenchymal differentiation. Moreover, compared to standard adult MSCs, they show enhanced immunomodulatory and anti‐fibrotic functions, and significantly extended lifespans in vitro for consistent quality in production. A recent report showed that intravenously delivered IMRCs could home into the lungs and inhibit both pulmonary inflammation and fibrosis after bleomycin‐induced acute lung injury in mouse models in vivo.^46^ Moreover, a pilot study for compassionate use of IMRCs showed that they could ameliorate the ARDS in two severely ill COVID‐19 patients. IMRCs’ hyper‐immunomodulatory function, pro‐regenerative paracrine signals and functional inhibition of TGF‐β1‐induced fibrosis were potential mechanisms for amelioration of pulmonary injury. Clinical trials for IMRCs are now in progress as well.

### 3.2 Immunomodulatory function of MSCs

MSCs have been widely used in basic research and clinical studies on immune‐mediated inflammatory diseases, such as graft‐versus‐host disease (GvHD), Crohn's disease, inflammatory bowel disease, rheumatoid arthritis and ARDS. MSCs’ properties in immunomodulatory and anti‐inflammatory signalling make them uniquely suited for these complex multifactorial diseases, including COVID‐19.^28^ As such, several clinical trials have launched for these diseases.^47^, ^48^ MSCs can activate immune regulatory responses through interactions with a wide repertoire of immune cells and participate in both innate immunity and adaptive immunity regulation.^49^ Below, we outline some of the host immune cells that MSCs interact with, either by direct contract or indirectly through paracrine secretion of various cytokines (Figure 1) to modulate the immune cells.^50^, ^51^

#### 3.2.1 T cells

MSCs broadly suppress T‐cell activation and proliferation in vitro via a plethora of soluble and cell contact‐dependent mediators. These mediators may act directly upon T cells or indirectly via modulation of antigen‐presenting cells and other accessory cells.^52^ MSCs can decrease the secretion of IFN‐γ and TNF‐α of T cells, and upregulate the secretion of IL‐4, so that the cells are transformed from a pro‐inflammatory state to an anti‐inflammatory state. In addition, MSCs can inhibit abnormally activated Th1 cells, restore the Th1/Th2 balance inhibit excessive proliferation of T cells and suppress the activity of cytotoxic CD8^+^ T lymphocytes via the NKG2D pathway.^53^ Thus, MSCs can improve the immune status by regulating the function of multiple subtypes of T cells.^54^, ^55^ Lymphopenia is also a prominent feature of COVID‐19, suggesting that SARS‐CoV‐2 infection causes T‐cell imbalance.^56^, ^57^, ^58^, ^59^ In fact, in severely ill COVID‐19 patients, the number of CD4^+^ and CD8^+^ T cells in the peripheral blood is often significantly reduced, while the overall immune system is abnormally activated and dysregulated by the cytokine storm during ARDS, suggesting a severe immune imbalance. Therefore, the immunomodulatory effects of MSCs and IMRCs on T cells may have potential therapeutic significance for patients with COVID‐19 and ARDS.

#### 3.2.2 Antigen‐presenting cells

MSCs can interfere with the antigen presentation functions, differentiation and maturation of dendritic cells (DC), thereby reducing DC activation and inflammatory factor secretion.^60^ MSCs regulate the differentiation of CD11c + B220‐DC precursors into regulatory DCs via prostaglandin E2 and PI3K signalling.^61^ MSCs vigorously promote the proliferation of mature DCs and drive mature DCs to transdifferentiate into a novel regulatory DC population to escape their apoptotic fate.^62^ In addition, MSCs can prevent DCs from secreting IFNγ and promoting T‐cell expansion in tumours.^63^ Since SARS‐CoV‐2 infection also results in DC reduction,^64^ MSCs could rescue DCs for the treatment of COVID‐19.

Macrophages are the other major antigen‐presenting cell type, and they are one of the cell types considered to play an essential role in ARDS.^65^ MSCs can regulate macrophage polarization via secretory exosomes to suppress chronic inflammation and promote tissue healing after injury.^66^ MSCs also secrete the TSG‐6 factor and IL‐10 to inhibit NF‐κB signalling and other pro‐inflammatory pathways, thereby driving the polarization of pro‐inflammatory M1 macrophages into anti‐inflammatory M2 macrophages.^54^, ^67^ Thus, MSCs could regulate macrophage polarization and their related signalling molecules, to modulate ARDS, the anti‐viral immunity, and tissue healing in COVID‐19 patients.^68^

#### 3.2.3 Neutrophils

Neutrophils can kill pathogens (bacteria, fungi and viruses) through an oxidative burst of reactive oxygen species (ROS) and phagocytosis, and they are recruited early to sites of infection to perform their defensive functions.^69^ Paradoxically, it has been reported that excessive neutrophil recruitment might exacerbate COVID‐19 immunopathology.^70^ Clinical studies have found that the number of neutrophils in the bronchoalveolar lavage fluid of ARDS patients is positively correlated with the severity of COVID‐19 and the cytokine storm.^71^ In fact, the neutrophil to lymphocyte ratio (NLR) can be used as an independent risk factor for severe disease in COVID‐19 patients.^72^ Intravenous injection of bone marrow mesenchymal stem cells (BMSCs)‐derived exosomes into severe COVID‐19 patients with ARDS can significantly reduce the production of neutrophils by 32%, thereby reducing their NLR levels and improving their clinical oxygenation index.^73^

#### 3.2.4 Other immune cells

MSCs can also inhibit excessive proliferation of B cells, prevent their differentiation into plasma cells and reduce excessive levels of immunoglobulin secretion by downregulating the expression of Blimp‐1.^55^ After MSC treatment, overactivated CXCR3^+^ NK cells also disappear in 3‐6 days, showing that MSCs have a potential regulatory effect on NK cells as well.^74^

#### 3.2.5 Tissue repair and regeneration capabilities of MSCs

Severely ill COVID‐19 patients often present severe pneumonia, respiratory failure, ARDS and pulmonary fibrosis. During this complex inflammatory pathogenic process,^55^, ^56^ the integrity of the lung alveolar capillary membrane is gradually destroyed, contributing to the formation of pulmonary oedema, lung tissue degeneration and fibrosis. MSCs can secrete a variety of growth factors and cytokines to improve the microenvironment of the lung tissue and promote endogenous lung repair, with potential benefits for COVID‐19 patients (Figure 2). For example, MSCs can promote cell proliferation and tissue damage repair by secreting hepatocyte growth factor (HGF), vascular endothelial growth factor (VEGF), keratinocyte growth factor (KGF) and fibroblast growth factor (FGF).^75^ MSCs secrete HGF through extracellular vesicles, reduce inflammatory damage and increase autophagy, thereby retaining or restoring the alveolar epithelium and the pulmonary vascular endothelial lining.^76^, ^77^ The HGF, KGF and angiopoietin‐1 secreted by MSCs also possess pro‐angiogenic, anti‐inflammatory and pro‐proliferation effects.^42^ It has been reported that MSCs can reduce apoptosis of the alveolar epithelial cells and endothelial cell by secretion these three growth factors.^77^, ^78^, ^79^ The VEGF and FGF secreted by MSCs can also promote lung tissue repair.^80^, ^81^

In addition, MSCs can reduce the levels of pro‐fibrotic factors to improve the microenvironment of lung cells and prevent pulmonary fibrosis, especially in patients with COVID‐19. One possible mechanism for this effect is exosome regulation. Exosomes are membrane‐bound extracellular vesicles of nanometre size (70‐150 nm), which not only participate in the communication between cells, but also participate in tissue damage repair.^82^ It has been demonstrated that MSCs and their exosomes (MSC‐Exo) considerably improved lung inflammation and pathological damage resulting from different types of lung injuries. MSC exosomes can promote the regeneration of alveolar epithelial cells, exert anti‐alveolar inflammation, prevent endothelial cell apoptosis, inhibit early epithelial‐mesenchymal trans‐differentiation and prevent myofibroblast growth by reducing the levels of TGF‐β, TNF‐α, type I collagen, type III collagen, hydroxyproline and serum ceruloplasmin in lung tissues, thereby alleviating pulmonary fibrosis.^83^ The MSC‐like IMRCs could also reverse pulmonary fibrosis by overexpressing the matrix metalloproteinase MMP1 and reducing collagen I levels during fibrogenesis induced by TGF‐β1.^84^

MSCs can also promote the repair of other damaged tissues in COVID‐19 patients. Pathological results show that SARS‐CoV‐2 virus can also affect the kidneys, causing severe acute tubular necrosis.^85^ Studies have shown that MSCs secrete cytokines to activate a variety of repair mechanisms in acute kidney injury, including anti‐inflammatory, anti‐apoptotic and pro‐angiogenic pathways, thereby promoting the repair of kidney injury.^86^, ^87^ MSCs can also treat COVID‐19‐related intestinal injury through mucosal repair and epithelial regeneration.^88^

## 4 STEM CELL THERAPY FOR COVID‐19 IN THE CLINIC

### 4.1 Guiding principles and management methods in China

According to '*The expert opinion for the prevention and therapy of novel coronavirus pneumonia'* guidelines, the clinical use of stem cell therapies for COVID‐19 is still in the exploratory stage in China. Treatments should be carried out only within the scope of the emergency project managed by the Ministry of Science and Technology of the People's Republic of China, in accordance with the '*The Expert Guidance on Clinical Research and Application of Stem Cell Therapy for Novel Coronavirus Pneumonia (COVID‐19)'* guidelines. Clinical research and clinical trials must be performed according to '*The Stem Cell Clinical Research Management Methods'* guidelines jointly issued by the National Health Protection Commission and the National Medical Products Administration in 2015, and '*The Guidelines for Quality Control and Preclinical Study of Stem Cell Preparations (Trial)'* to ensure stem cell therapies for COVID‐19 are tested in a scientifically and medically rigorous manner consistent with internationally accepted standards.^89^

For example, researchers are required to work out a detailed clinical research plan and pass the scientific review of the academic committee of clinical research institutions and the ethical review of the ethics committee. All participating units should carry out clinical research under the conditions of compliance with ethics, informed consent, project filing and clinical registration. Moreover, the preparation of stem cells must be carried out according to '*The Guidelines for Quality Control and Preclinical Study of Stem Cell Preparations (Trial)'*, and the quality of stem cells must meet the required standards for human clinical trials of stem cell drugs and receive official approval from the National Medical Products Administration before clinical trials can be initiated.

More specifically, indications allowed for stem cell therapy in COVID‐19 include severe or critical illness from COVID‐19‐related pneumonia. Patients should receive no more than 3 rounds of stem cell infusion. The dose of stem cell injection for each round should be 1 to 5 × 10^6^ cells/kg body weight, and the interval time between each round is recommended to be no less than 3 days. A proper clinical research programme must be designed according to the specific goals of the clinical research and the actual working conditions for clinical implementation. Multi‐centre, randomized controlled and double‐blinded trials are recommended. Patients in both the stem cell treatment arm and the control arm should receive conventional treatments recommended by the above guidelines. The placebo used in the control arm should contain only normal saline plus human serum albumin without stem cells. Follow‐up after treatment is strictly required according to the clinical protocol guidelines.

### 4.2 Clinical trials for COVID‐19 stem cell therapies

#### 4.2.1 Overview

Clinical trials for stem cell therapies against COVID‐19 were searched by using the terms 'COVID‐19' and 'stem cells' in the ClinicalTrials.gov database (https://clinicaltrials.gov), the World Health Organization International Clinical Trials Registry Platform (Chinese Clinical Trial Registry, http://www.chictr.org.cn) and the European Union Clinical Trials Register (https://www.clinicaltrialsregister.eu) (September 2020) (Table S1). All observational studies and 6 withdrawn clinical studies were excluded from the list. Eventually, 88 clinical trials related to stem cells were found to be registered in different countries. In these clinical studies, the therapeutic efficacy (60 trials) and the safety (32 trials) of stem cells and their derivatives for treating COVID‐19 were being investigated.

#### 4.2.2 Indications and phases

**FIGURE 3:**
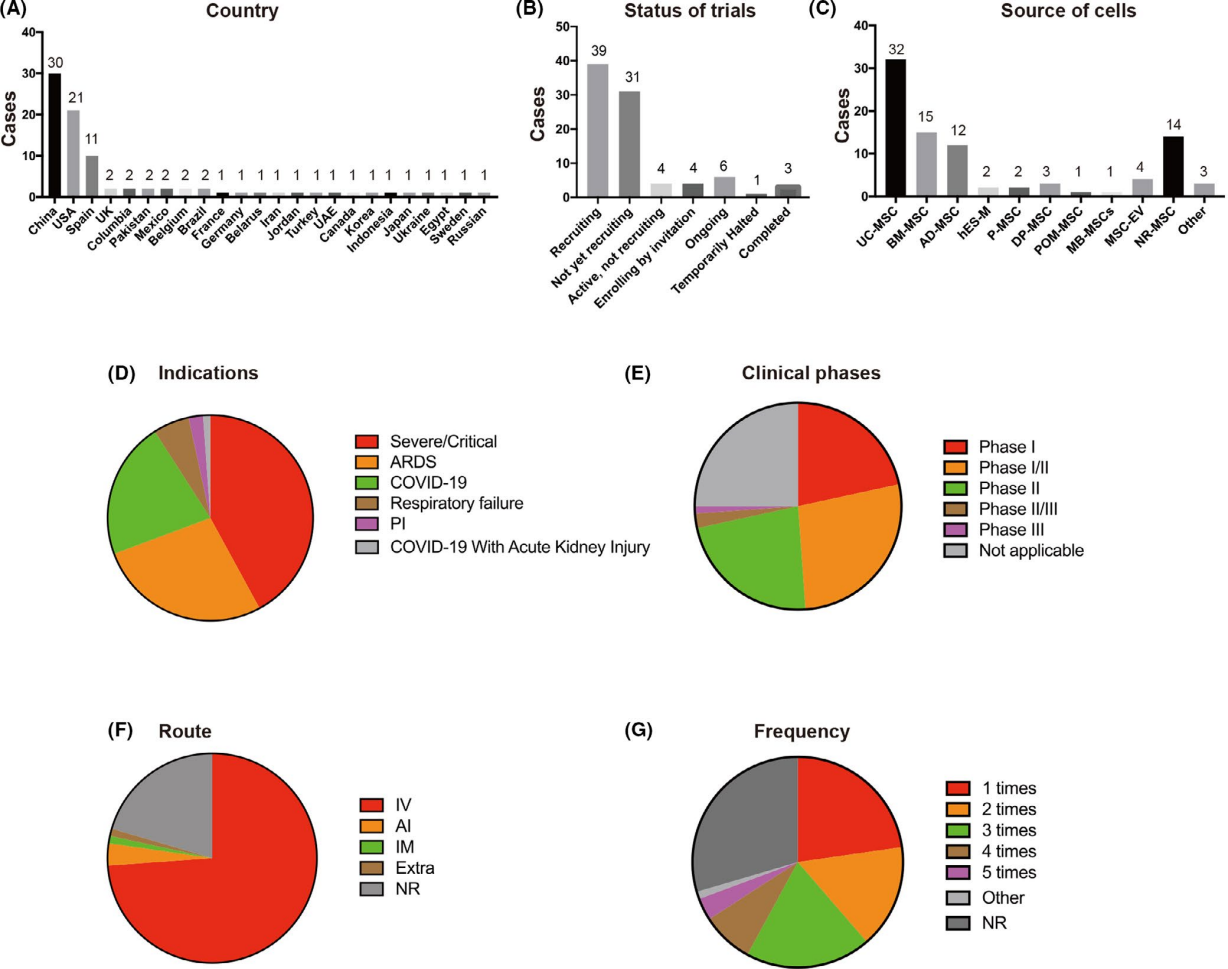
Statistical summary of countries, status of trials, source of cells, indications, clinical phases, administrative routes and frequency of doses used by stem cell therapies against COVID‐19. UC‐MSCs, umbilical cord‐derived MSCs; BM‐MSCs, bone marrow‐derived MSCs; AD‐MSCs, adipose‐derived MSCs; hES‐M, MSC‐like cells derived from human embryonic stem cells; P‐MSCs, placenta‐derived MSCs; DP‐MSC, dental pulp‐derived MSCs; MSC‐EV, extracellular vesicles derived from MSCs; POM‐MSC, pooled olfactory mucosa MSCs; MB‐MSCs, menstrual blood‐derived MSCs; ARDS, acute respiratory distress syndrome; PI, pulmonary interstitial fibrosis; IV, intravenous; IM, intramuscular; AI, aerosol inhalation; Extra, extracorporeal stromal cell therapeutic; NR, not reported

In total, 88 trials were found to be registered to investigate the safety and efficacy of transplantation therapy of stem cells or stem cell‐derived exosomes for COVID‐19 patients. Indications under investigation include COVID‐19 with severe/critical pneumonia, respiratory failure, ARDS and pulmonary fibrosis (Figure 3). Most studies were registered to treat patients with 'COVID‐19' (19 out of 88) and 'severe/critical pneumonia' (37 out of 88). According to a meta‐analysis of 50 466 hospitalized patients with COVID‐19, 14.8% of COVID‐19 patients developed ARDS.^90^ Treatment of patients with 'ARDS' was being investigated in 24 of 88 studies. Although ARDS patients often manifest pulmonary fibrosis after hospital discharge,^90^, ^91^ only 2 of 88 studies were registered to investigate the efficacy of stem cell therapies in patients with 'pulmonary fibrosis', one of which uses 'MSCs derived from human embryonic stem cells' (ChiCTR2000031139). Interestingly, only 1 of 88 studies is using 'extracorporeal stromal cell therapeutics' to treat COVID‐19 patients with acute kidney injury (NCT04445220). The vast majority of these clinical trials (63 out of 88) are testing the safety of stem cell therapies for feasibility, tolerance, and severe adverse events (19 trials for Phase I, 24 for Phase I/II, and 20 for Phase II). Few clinical trials have progressed beyond Phase II (3.4%), with only 2 trials in Phase II/III and only 1 trial in Phase III. In 22 studies, the clinical phase is unclear or 'Not Applicable' (Figure 3).

#### 4.2.3 Country and status

Because COVID‐19 was first officially reported in China, it is unsurprising that 34% of these trials were registered in China. In China, a total of 30 clinical studies have been registered, with 18 clinical trials in the Chinese Clinical Trial Registry and 12 clinical trials in the US National Institutes of Health (Figure 3A). One clinical trial has been completed, and 16 trials are still 'Recruiting'. In China, strict quarantine rules and epidemic control measures led to rapid suppression of the COVID‐19 outbreak before the summer of 2020. As a result, many registered clinical trials could not be completed due to insufficient patients, and 13 clinical trials are 'Not yet recruiting'. Numerous other countries like the United States (21 out of 88), Spain (11 out of 88), UK (2 out of 88), France (1 out of 88), Columbia (2/88), Belgium (2 out of 88), Brazil (2 out of 88), Mexico (2 out of 88) and Pakistan (2/88) are also carrying out clinical trials for stem cell therapies against COVID‐19 (Figure 3A). Globally, the clinical trials are mainly concentrated in China, the United States and Europe, which may be related to the severity of the epidemic and the strength of their scientific research infrastructure. Most of these studies are 'Recruiting' or 'Ongoing' (29 out of 58), although 3 clinical trials have been completed (Figure 3B).

#### 4.2.4 Source and dosing of stem cells

The source of stem cells used in these trials is a major point of variability amongst the searched studies (Figure 3C). Because of the aforementioned advantages of MSCs in immune regulation and tissue repair, it is not surprising that MSCs have become the predominant stem cell type in COVID‐19 clinical trials. The most common source of MSCs was the umbilical cord/Wharton's jelly (32 out of 88), followed by bone marrow (15 out of 88), adipose tissue (12 out of 88), dental pulp (3 out of 88), placenta (2 out of 88) and menstrual blood (1 out of 88). In 14 studies, the MSCs source was unclear. Interestingly, in 2 studies, human embryonic stem cell‐derived MSCs (hES‐M) were being used (ChiCTR2000031139, NCT04331613), likely due to the indefinite expansion potential and consistent quality associated with embryonic stem cells. Considering the limited expansion potential of adult MSCs, extracellular vesicles derived from MSCs (MSC‐EV) were also being used as an alternative in 4 studies. Pooled olfactory mucosa MSCs were also used in 1 study (NCT04382547). Finally, only 3 studies used non‐MSCs, namely cord blood stem cells, autologous immune cells and autologous non‐hematopoietic peripheral blood stem cells (NHPBSCs).

Cell dose and proposed regimens also varied greatly amongst studies. While the MSC infusion dosage ranged over an order of magnitude between 0.5 × 10^6^ and 10 × 10^6^ cells/kg (Table S1), the most commonly used infusion dosage was 1 × 10^6^ cells/kg. In some studies, stem cells are infused regardless of the weight of the patient. In these cases, the proposed infusion dosage ranged from 1.5 × 10^7^ to 75 × 10^7^ cells per round regardless of the weight of the patient, with 10 × 10^7^ cells per round as the most common dose (Table S1). The highest dose of 75 × 10^7^ MSCs per round was used for the 'extracorporeal stromal cell therapeutics' against COVID‐19‐related acute kidney injury (NCT04445220). Of note, higher cell doses will likely bring higher treatment risks. Therefore, it is necessary to find a balance between therapeutic efficacy and safety concerns.

#### 4.2.5 Route and frequency of administration

Intravenous injection of MSCs may produce a first‐order lung effect,^92^ which leads to significant cell retention in the lungs, thus providing an advantage for lung tissue repair in COVID‐19, ARDS and pulmonary fibrosis. Therefore, most of the ongoing clinical trials proposed to perform intravenous cell infusion (65 out of 88; Figure 3F). Three studies focused on the administration of MSCs‐derived exosomes via the inhalation route. Intramuscular injection of MSCs was used in one study (NCT04389450). The 'extracorporeal stromal cell therapeutics' was used in COVID‐19 subjects with acute kidney injury in a study (NCT04445220). However, in 18 studies, the route of MSCs administration was not clearly stated.

Although a single round of MSC infusion, as proposed in 20 out of 88 trials, has been shown to provide therapeutic benefits, more than one round may be required to induce complete tissue repair or even to maintain therapeutic benefits (Figure 3G). In some studies, the mentioned MSC doses would be injected two (14 out of 88), three (17 out of 88), four (7 out of 88) and even five rounds (3 out of 88) with short time intervals of 2 or 3 days (Figure 3G; Table S1).

### 4.3 The safety and efficacy of stem cell therapy in COVID‐19

Recently, some studies have been published to report the safety and efficacy of stem cell therapy for COVID‐19 (Table 1). In these published reports, MSCs derived from the umbilical cord (UC), adipose tissue (AD), bone marrow (BM), menstrual blood (MB), dental pulp (DP), human embryonic stem cells (hESCs) and exosomes derived from MSCs were used. While several biotech companies including Athersys, Cynata, Mesoblast and Pluristem have also initiated clinical trials with MSC‐like cells for COVID‐19, some with announcements of preliminary success, we will not cover them below because not all the details of their clinical trials are available yet.

**TABLE 1:** Published clinical trials of stem cell therapies against COVID‐19

Trial ID no.	Indications	Patient population	Source of cells	Dose of cells	Route	Frequency	Clinical phases	Reference
ChiCTR2000029990^a^	Moderate/Severe/Critical	7	MSCs	1 × 10^6^ cells/kg/round	IV	1 round	Phase I/II	74
NR	Severe/Critical	25	MSCs	1 × 10^6^ cells/kg/round	IV	2 or 3 rounds (interval 5 d)	NR	97
NCT 03042143^b^	ARDS	60	UC‐MSCs	40 × 10^7^ cells/round	IV	1 round	Phase I/II	112
ChiCTR2000031319 NCT04336254	Severe/Critical	20	DP‐MSCs	3 × 10^7^ cells/round	IV	3 rounds at day 1, 4 and 7	Phase I/II	113
NCT04331613	ARDS	2	hESC‐IMRCs (CAStem)	3, 5, 10 × 10^6^ cells/kg/round	IV	1 round	Phase I	84
NR	ARDS	24	BM‐MSCs‐EV	15 mL	IV	1 round	NR	73
NCT04348461	Severe/Critical	13	AD‐MSCs	1 × 10^6^ cells/kg/round	IV	1, 2 or 3 rounds	Phase II	101
ChiCTR2000029606	ARDS	2	MB‐MSCs	NR	IV	3 rounds	Not Applicable	106
ChiCTR2000031494	Severe/Critical	41	UC‐MSCs	2 × 10^6^ cells/kg/round	IV	1 round	Phase I	98
NR	Severe/Critical	1	UC‐MSCs	5 × 10^7^ cells/round	IV	3 rounds (interval 3 d)	NR	99
NCT04288102	Severe/Critical	18	UC‐MSCs	3 × 10^7^ cells/round	IV	3 rounds (on days 0, 3 and 6)	Phase I	100

Abbreviations: MSCs, mesenchymal stem cells; UC‐MSCs, umbilical cord‐derived MSCs; BM‐MSCs, bone marrow‐derived MSCs; AD‐MSCs, adipose‐derived MSCs; DP‐MSCs, dental pulp‐derived MSCs; BM‐MSCs‐EV, exosomes derived from bone marrow‐derived MSCs; IV, intravenous; MB‐MSCs, menstrual blood‐derived MSCs; hESC‐IMRCs, immunity‐ and matrix‐regulatory cells from human embryonic stem cells; NR, not reported.

^a^ http://www.chictr.org.cn

^b^ https://clinicaltrials.gov/

#### 4.3.1 MSCs

The first study for stem cell treatment of COVID‐19 by Dr Zhao (ChiCTR2000029990)^74^ reported that intravenous administration of clinical‐grade human mesenchymal stem cells (MSCs) into 7 COVID‐19 patients resulted in improved functional outcomes and facilitated recovery. In this study, 7 enrolled patients (1 critical illness, 4 severe illness, 2 moderate illness) received intravenous infusions of MSCs. After 14 days, MSCs could significantly improve the functional outcomes of all 7 patients without any adverse effects observed. The pulmonary function and symptoms of these 7 patients were significantly improved within 2 days after MSC transplantation. After treatment, the peripheral lymphocytes were increased, the C‐reactive protein decreased, and the overactivated cytokine‐secreting immune cells disappeared in 3‐6 days. Moreover, their gene expression profile showed that MSCs were negative for both ACE2 and TMPRSS2, which are required by the SARS‐CoV‐2 virus to enter host cells,^93^, ^94^, ^95^, ^96^ thus suggesting MSCs do not carry risks for SARS‐CoV‐2 cross‐contamination.

Another retrospective study evaluated the treatment efficacy and side effects of MSC therapy on severe COVID‐19.^97^ In total, 25 patients were enrolled according to their inclusion/exclusion criteria. A total of 7 patients received 1 round, 7 patients received 2 rounds, and 11 patients received 3 rounds of MSCs therapy. After MSC therapy, 16 patients (64%) showed improvements by chest CT scans and all patients showed clinical improvements. No fatalities occurred during hospitalization. However, no significant changes in inflammation indices, IgG and IgM were found, and the serum levels of lactate (LAC), cardiac troponin T (cTnT) and creatine kinase MB (CK‐MB) elevated significantly after MSC therapy, suggesting little improvement in immunomodulation and some cardiotoxicity after MSC therapy. While the reasons are unclear, possibly related to a mild cytokine storm, missing the optimal detection time window and limited analysis of inflammation indices, these results serve as a reminder that stem cell clinical trials should be extremely cautious on patients with underlying metabolic diseases and their inflammatory marker analyses.

##### Umbilical cord‐derived MSCs (UC‐MSCs)

Shu et al reported that infusion of UC‐MSCs was effective and safe for the treatment of severe COVID‐19 (ChiCTR2000031494).^98^ In this study, 12 COVID‐19 patients with severe illness received intravenous infusions of UC‐MSCs. Results showed that the 28‐day mortality rate was zero in the UC‐MSCs treatment arm, whereas the mortality rate was 10.34% in the control arm. After MSCs transplantation, the time to clinical improvement was shorter than that in the control arm. UC‐MSC infusion improved clinical symptoms, including weakness and fatigue, shortness of breath, and low oxygen saturation. UC‐MSCs could reduce inflammatory CRP and IL‐6 levels, accelerate the recovery of lymphocyte count and shorten the lung inflammation absorption period. Intravenous UC‐MSCs was found to be a safe and effective treatment option for severe COVID‐19.

Liang et al reported a case report of a critically ill COVID‐19 patient treated by UC‐MSCs.^99^ The patient received 3 rounds of allogeneic UC‐MSCs (5 × 10^7^ cells each round), together with thymosin a1 and antibiotics via daily injection. UC‐MSC infusion improved most of the laboratory indices and relieved the inflammation symptoms. Throat swab tests for SARS‐CoV‐2 turned negative on both day 21 and day 23. On day 30, the previously critically ill patient was discharged from hospital after recovery.

Wang et al reported a Phase I clinical trial of UC‐MSCs for COVID‐19 (NCT04288102).^100^ COVID‐19 patients with moderate and severe pulmonary disease received 3 rounds of intravenous infusions of UC‐MSCs (3 × 10^7^ cells per infusion) on days 0, 3 and 6, respectively. No serious infusion‐associated adverse events were observed. In most severe patients, the PaO_2_/FiO_2_ ratio improved after UC‐MSC treatment. In the UC‐MSC treatment arm, 2 moderate and 2 severe patients with high baseline IL‐6 levels showed a decrease in IL‐6 within 3 days after UC‐MSC infusion and remained stable in the following 4 days. Chest CT scans showed that the lung lesions of patients receiving UC‐MSC infusions were well controlled within 6 days, and completely disappeared within 2 weeks after UC‐MSC infusion. In the control arm, 1 severe patient still had obvious pulmonary lesions at the point of discharge.

##### Adipose tissue‐derived MSCs

Sanchez‐Guijo et al reported a study of Adipose tissue‐derived MSCs (AD‐MSCs) for the treatment of patients with severe COVID‐19‐related pneumonia (NCT04348461).^101^ A total of 13 severe COVID‐19 pneumonia patients under mechanical ventilation received intravenous administration of AD‐MSCs. No adverse events related to cell therapy were observed. Clinical improvement was observed in 9 patients (69.2%), 7 patients were extubated and discharged from ICU, and 4 patients remained intubated. Administration of AD‐MSCs reduced the levels of inflammatory markers C‐reactive protein, IL‐6, ferritin, LDH and D‐dimer, and increased the lymphocyte counts.

##### Menstrual blood‐derived MSCs

In 2007, MSCs were identified in the endometrial tissues shed along with menstrual blood.^102^ Since then, this subtype of MSCs has been studied in basic medical research and clinical trials.^103^, ^104^, ^105^ Li et al reported a clinical study using Menstrual blood‐derived MSCs (MB‐MSCs) for the treatment of COVID‐19 patients with severe illness (ChiCTR2000029606).^106^ MB‐MSC transplantation increased CD4 + lymphocyte counts and decreased the levels of inflammatory markers IL‐6 and C‐reactive protein. After MB‐MSC transplantation, the oxygen saturation (SaO_2_) and partial pressure of oxygen (PO_2_) improved. Additionally, the chest CT scans indicated that the patients’ bilateral lung exudate lesions were adsorbed after MB‐MSC infusion.

##### MSC‐like stem cells derived from hESCs

Despite their safety and efficacy, clinical applications of primary MSCs derived from the umbilical cord, bone marrow or adipose tissue have been hampered by the lack of available donor tissues, limited cell numbers from each donor, donor and tissue heterogeneity, inconsistent cell quality and the lack of standardized cell preparations. Wu et al reported a cell population derived from hESCs and named it as immunity‐ and matrix‐regulatory cells (IMRCs, also called CAStem).^84^ IMRCs resembled MSCs in their capacity for self‐renewal and tri‐lineage mesenchymal differentiation, but displayed an even higher consistency in quality, even stronger immunomodulatory and anti‐fibrotic functions, and a robust ability to treat lung injury and fibrosis in vivo. On this basis, IMRCs were approved for compassionate use in a pilot study and subsequently a Phase I trial (NCT04331613), in response to the emergency of the COVID‐19 crisis in China. After patient consent, IMRCs were administered intravenously in 2 severely ill COVID‐19 patients. After IMRC transfusion, both the severely ill COVID‐19 patients showed significant recovery from pneumonia, tested negative for SARS‐CoV‐2 and were recommended for discharge within 14 days. Many pro‐inflammatory cytokines were suppressed after IMRC infusion, including GRO‐α, IFN‐α2, IL‐3, IL‐9, IL‐13, MCP‐3, M‐CSF, sCD40L and TNF‐α by day 4‐8. This is the first clinical trial for COVID‐19 treatment using hESC‐derived cells, and the preliminary results suggest both efficacy and at least short‐term safety in COVID‐19 patients with severe illness.

#### 4.3.2 Exosomes Derived from MSCs

MSCs transplantation is limited by safety, cell survivability, scalability, consistency and regulatory issues that make it difficult to meet the needs of millions of SARS‐CoV‐2 infected patients worldwide.^74^, ^99^, ^107^ Exosomes or extracellular vesicles (EVs) derived from MSCs present another potential option for the large numbers of COVID‐19 patients, since MSCs cultured in vitro can continuously shed large amounts of exosomes into the conditioned media instead of dying shortly after transplantation in vivo. Multiple preclinical studies have shown that exosomes from MSCs exert favourable therapeutic effects in animal models of acute lung injury (ALI), ARDS, fibrosis and other inflammatory diseases. Exosomes function by reducing inflammation, enhancing oedema clearance, promoting restoration of leaky epithelial membranes and reducing other sequelae of the cytokine storm.^108^, ^109^, ^110^, ^111^ Sengupta et al reported that exosomes (ExoFlo^TM^) derived from BM‐MSCs could work in severe COVID‐19.^73^ In this study, 24 SARS‐CoV‐2 PCR positive patients received a single 15 mL intravenous dose of ExoFlo derived from allogeneic BM‐MSCs. No adverse events were observed within 72 hours of ExoFlo administration. 83% of the patients survived, and 71% of the patients recovered. Moreover, patients’ clinical status and oxygenation index improved after just one treatment. Meanwhile, reductions of absolute neutrophil count and increases of absolute lymphocyte count were observed, that is, the NLR decreased. Likewise, ExoFlo reduced the inflammatory markers C‐reactive protein, Ferritin and D‐dimer. This study demonstrated that only a single intravenous dose of BM‐MSC‐derived exosomes could effectively and safely treat patients with severe COVID‐19. Indirectly, the clinical success of MSC‐derived exosomes also supports the idea that MSCs likely treat COVID‐19 pulmonary disease via a paracrine and secretory mechanism.

## 5 FUTURE PROSPECTS

Of all the stem cell therapies touted for COVID‐19 treatment, MSCs or MSC‐like derivatives have been the most promising in preclinical studies and clinical trials so far. Intravenously infused MSCs have been found to migrate directly to the lungs, where they can secrete numerous factors that play an important role in immunomodulation, protecting alveolar epithelial cells, resisting pulmonary fibrosis and improving lung function, which is a great benefit for treating severe pulmonary disease in COVID‐19. The main mechanism of their therapeutic effect is through the secretion of soluble factors, such as cytokines, chemokines, angiogenic factors, growth factors, and exosomes and extracellular vesicles. While multifactorial in nature, a large corpus of research work suggests that it is these complex mechanisms that make them suitable for treating complex and multifactorial diseases for which no other reductionistic drug treatments are available yet, such as COVID‐19‐related ARDS and other similar inflammatory diseases that involve a cytokine storm (CRS). The extraordinarily rapid global spread of SARS‐CoV‐2 and the rapidly escalating public health emergency of the COVID‐19 pandemic had essentially forced multiple institutions across the world to take the leap of faith and put multiple types of stem cell therapies into pilot studies and clinical trials. Fortunately, MSCs and MSC‐like derivatives have shown some promising results in safety and efficacy.

However, more work remains to be done. Although several clinical trials have preliminarily demonstrated the safety and efficacy of intravenous MSCs in patients with COVID‐19‐related lung diseases, the unclear heterogeneity of the sources of MSCs and thus their secretory and immunomodulatory capabilities make it difficult to compare and learn from the clinical trial results from different studies. Moreover, the consistency and uniformity of stem cell quality cannot be quantified nor guaranteed at this point. Therefore, overcoming the heterogeneity of stem cells is one of the most pressing issues of stem cell therapy in the clinic. Future work should focus on the development, dissemination and international agreement on clinical standards to quantify the quality and consistency of stem cell therapies, proper completion and publication of existing clinical trials for the COVID‐19 crisis, and the development of scalable technologies and resources for producing the large numbers of stem cells needed in a public health crisis. For nobody knows yet when the current COVID‐19 crisis will end, and when the next crisis will come.

## CONFLICT OF INTEREST

The authors declare no conflict of interest.

## AUTHOR CONTRIBUTIONS

YT, JW and JH conceived the project and supervised the manuscript. ZL, SN, B.G and TG contributed equally to this work and wrote the manuscript with help from all the authors. ZL, SN, BG, TG, LW, YW, L.W., YT, JW and JH participated in the experiments and data analysis.

## Supporting information

Table S1

## Data Availability

The data that support the findings of this study are available in the Table S1 of this article.
